# Tirzepatide on obstructive sleep apnea-related cardiometabolic risk: secondary outcomes of the SURMOUNT-OSA randomized trial

**DOI:** 10.1038/s41591-025-04071-1

**Published:** 2026-01-15

**Authors:** Atul Malhotra, Ronald Grunstein, Ali Azarbarzin, Scott Sands, Virend K. Somers, Louis J. Aronne, Ania M. Jastreboff, Jitong Lou, Sujatro Chakladar, Julia P. Dunn, Mathijs C. Bunck, Josef Bednarik

**Affiliations:** 1 University of California San Diego, La Jolla, CA USA; 2 Woolcock Institute of Medical Research, Macquarie University and Royal Prince Alfred Hospital, Sydney, New South Wales Australia; 3 Brigham and Women’s Hospital and Harvard Medical School, Boston, MA USA; 4 Department of Cardiovascular Medicine, Mayo Clinic, Rochester, MN USA; 5 Comprehensive Weight Control Center, Division of Endocrinology, Diabetes and Metabolism, Weill Cornell Medicine, New York, NY USA; 6 Yale University School of Medicine, New Haven, CT USA; 7 Eli Lilly and Company, Indianapolis, IN USA

**Keywords:** Phase III trials, Cardiovascular diseases, Metabolic disorders

## Abstract

Obstructive sleep apnea (OSA) is associated with obesity and cardiovascular risk. The SURMOUNT-OSA master protocol comprised two, 52-week, randomized, double-blind, placebo-controlled phase 3 studies (study 1 and study 2) and demonstrated a significant reduction of a number of cardiometabolic risk measures in participants with OSA and obesity following treatment with tirzepatide. Here we report prespecified analysis of cardiometabolic risk measures in SURMOUNT-OSA. Post hoc analyses include changes in a homeostatic model assessment for insulin resistance and mediation analysis to determine the proportion of observed changes attributable to reductions in body weight, apnea–hypopnea index and sleep apnea-specific hypoxic burden. In both study 1 and study 2 of SURMOUNT-OSA, tirzepatide treatment was associated with greater alleviation of cardiometabolic risk factors than placebo. Independent mediation effect of changes in OSA metrics was observed on high-sensitivity C-reactive protein, homeostatic model assessment for insulin resistance and triglycerides. The combination of changes in weight and OSA metrics, as well as weight alone, had a significant mediation effect on systolic blood pressure, but there was no significant mediation effect of weight or OSA metrics observed on diastolic blood pressure. Based on the mediation analysis, treating both sleep-disordered breathing and obesity is likely required to optimize the treatment effect on cardiometabolic benefits for patients with moderate-to-severe OSA and obesity. The ClinicalTrials.gov registration number for this study is NCT05412004.

## Abstract (web summary)

In a prespecified secondary analysis of the SURMOUNT-OSA trial, tirzepatide improved cardiometabolic outcomes in patients with moderate-to-severe obstructive sleep apnea and obesity.

## Main

Obstructive sleep apnea (OSA) is a common disorder with major cardiometabolic and neurocognitive sequelae^1^. Excess adiposity is a major reversible etiologic risk factor for OSA and its complications^2,3^. Nasal continuous positive airway pressure (PAP) to this point has been first line therapy for OSA but is not always well tolerated^4,5^. Moreover, PAP has not shown consistent benefits in patients with OSA from the perspective of cardiovascular outcomes^6^. The reason for these findings is unclear but may relate to underlying risk factors such as obesity, which are not directly addressed by PAP^7^.

Despite advances in obesity pharmacotherapy, the obesity epidemic is predicted to continue to increase over time, leading to a rising prevalence of OSA^8,9^. Weight reduction in patients with OSA and obesity yields notable improvements in OSA severity measured by apnea–hypopnea index (AHI)^9,10^. Considerable interest has been accumulated regarding the use of tirzepatide, a glucose-dependent insulinotropic polypeptide (GIP) and glucagon-like peptide-1 (GLP-1) receptor agonist, which has previously been shown to improve body weight as well as glucose control^11,12^. The results of the SURMOUNT-OSA program studies were recently published, demonstrating tirzepatide treatment to be superior to placebo in participants not using PAP therapy (study 1) as well as those using PAP therapy (study 2)^13^. The primary outcome of the SURMOUNT-OSA studies was the change in AHI, which is the traditional marker of sleep apnea severity^13^. However, the studies also reported key secondary outcomes that were controlled for multiplicity including high-sensitivity C-reactive protein (hsCRP), systolic blood pressure (SBP), sleep apnea-specific hypoxic burden (SASHB) and body weight, as well as patient reported outcomes (PROMIS)^13^. The results lead to questions about how much of the cardiometabolic benefits associated with tirzepatide are related to improvement in OSA severity per se versus improvement in body weight. In addition, other cardiometabolic outcomes were not reported, leading to questions about the overall benefits of tirzepatide treatment on patients with OSA and obesity^13^.

Based on this conceptual framework and including a complete set of study prespecified cardiometabolic endpoints, we sought to test the hypothesis that tirzepatide treatment in the SURMOUNT-OSA studies would yield improvement in cardiometabolic risk measures as compared to placebo. We include the previously reported effects of tirzepatide on hsCRP, systolic and diastolic blood pressure (DBP)^13^. We report the effects of tirzepatide on high-density lipoprotein-cholesterol (HDL-C), non-HDL-C and fasting insulin that were prespecified in the study protocol^14^. We further report the effects of tirzepatide on low-density lipoprotein-cholesterol (LDL-C), very low-density lipoprotein-cholesterol (VLDL-C) and homeostatic model assessment for insulin resistance (HOMA-IR) and a mediation analysis in the pooled sample of SURMOUNT-OSA participants, which seeks to determine how much of any observed improvement in analyzed cardiometabolic risk measures is related to changes in OSA severity factors versus changes in weight versus interaction between these variables.

## Results

### Participants

**Fig. 1:**
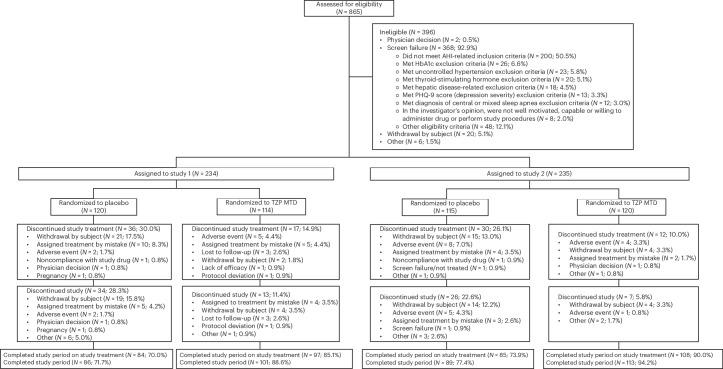
Study design flowchart. The data from a previously published study protocol^13^. HbA1c, hemoglobin A1C; MTD, maximum tolerated dose; TZP, tirzepatide. Source data

A total of 469 participants were randomized to receive either tirzepatide or placebo across study 1 (Fig. 1; 234 participants; tirzepatide *n* = 114, placebo *n* = 120) and study 2 (Fig. 1; 235 participants; tirzepatide *n* = 120, placebo *n* = 115). The full participant disposition can be found in Fig. 1. The baseline characteristics of investigated cardiometabolic risk measures were, in general, comparable between the tirzepatide and placebo groups across both study 1 and study 2 at baseline. The details of the baseline demographics and clinical characteristics can be found in Table 1, some of which have been previously reported^13^.

**Table 1:** Baseline demographics and clinical characteristics

Characteristic	Study 1			Study 2		
	Tirzepatide MTD (*n* = 114)	Placebo (*n* = 120)	Total (*n* = 234)	Tirzepatide MTD (*n* = 120)	Placebo (*n* = 115)	Total (*n* = 235)
Age, years	47.3 ± 11.0	48.4 ± 11.9	47.9 ± 11.5	50.8 ± 10.7	52.7 ± 11.3	51.7 ± 11.0
Female, *n* (%)	36 (31.6)	41 (34.2)	77 (32.9)	33 (27.5)	32 (27.8)	65 (27.7)
BMI, kg m^−^^2^	39.7 ± 7.3	38.6 ± 6.7	39.1 ± 7.0	38.6 ± 6.1	38.7 ± 6.0	38.7 ± 6.0
AHI, events per hour	52.9 ± 30.5	50.1 ± 31.5	51.5 ± 31.0	46.1 ± 22.4	53.1 ± 30.2	49.5 ± 26.7
OSA severity, *n* (%)						
No apnea	0 (0.0)	1 (0.8)	1 (0.4)	–	–	–
Mild	1 (0.9)	2 (1.7)	3 (1.3)	0 (0.0)	2 (1.8)	2 (0.9)
Moderate	39 (34.2)	43 (36.1)	82 (35.2)	35 (29.4)	37 (32.5)	72 (30.9)
Severe	74 (64.9)	73 (61.3)	147 (63.1)	84 (70.6)	75 (65.8)	159 (68.2)
SASHB, %min h^−1^ (CV (%))†	153.6 (102.7)	137.8 (104.1)	145.3 (103.4)	132.2 (83.4)	142.1 (112.5)	137.0 (97.5)
SBP, mmHg	128.2 ± 11.9	130.3 ± 10.8	129.3 ± 11.4	130.7 ± 14.2	130.5 ± 12.8	130.6 ± 13.5
DBP, mmHg	83.7 ± 8.9	83.9 ± 8.7	83.8 ± 8.8	83.2 ± 8.1	80.5 ± 8.6	81.9 ± 8.5
hsCRP, mg l^−1^ (CV (%))†	3.6 (120.0)	3.8 (110.7)	3.7 (115.1)	3.0 (124.2)	2.7 (123.4)	2.8 (123.6)
HDL-C, mmol l^−1^ (CV (%))†	1.1 (23.3)	1.2 (23.4)	1.1 (23.4)	1.1 (20.8)	1.2 (27.3)	1.1 (24.1)
Non-HDL-C, mmol l^−1^ (CV (%))†	3.8 (29.6)	3.7 (24.3)	3.7 (27.1)	3.8 (24.0)	3.5 (26.1)	3.7 (25.2)
Triglycerides, mmol l^−1^ (CV (%))†	1.7 (59.8)	1.7 (51.0)	1.7 (55.4)	1.7 (43.5)	1.7 (52.2)	1.7 (47.5)
Fasting insulin, mU l^−1^ (CV (%))†	22.8 (89.0)	17.9 (60.0)	20.3 (76.5)	17.9 (64.9)	20.8 (72.9)	19.1 (69.1)
HbA1c (%)	5.7 ± 0.4	5.6 ± 0.4	5.7 ± 0.4	5.6 ± 0.4	5.7 ± 0.4	5.6 ± 0.4
HOMA-IR (CV (%))†	5.6 (104.7)	4.4 (63.8)	5.0 (86.3)	4.5 (67.1)	5.1 (80.1)	4.7 (73.4)
Hypertension, *n* (%)	84 (73.7)	93 (77.5)	177 (75.6)	91 (75.8)	91 (79.1)	182 (77.4)
Prediabetes, *n* (%)	74 (64.9)	78 (65.0)	152 (65.0)	69 (57.5)	64 (55.7)	133 (56.6)

The table contains some previously reported data^13^.

The data are the mean ± s.d. unless otherwise specified.

†Data are geometric means (coefficient of variation, in percentage).

BMI, body mass index; CV, coefficient of variation; *n*, number of randomized patients with at least one dose of study drug; MTD, maximum tolerated dose; s.d., standard deviation.

### Changes in cardiometabolic risk measures

The SURMOUNT-OSA statistical analysis plan prespecified assessment of changes in SBP and DBP, hsCRP, HDL-C, non-HDL-C, LDL-C, VLDL-C and fasting insulin. The changes in HOMA-IR were analyzed post hoc.

Changes in SBP and DBP were assessed at week 48 to prevent confounding effect of PAP discontinuation at week 52 in study 2, all other changes were assessed at week 52.

There was a significant estimated treatment difference (ETD) between tirzepatide and placebo for change in SBP from baseline at week 48 both in study 1 (−7.9 mmHg; 95% confidence interval (CI) −11.0 to −4.9; *P* < 0.001) and in study 2 (−4.3 mmHg; 95% CI −7.3 to −1.2; *P* = 0.007). The ETD between tirzepatide and placebo of change in DBP from baseline at week 48 was significant in study 1 (−3.2 mmHg; 95% CI −5.4 to −1.0; *P* = 0.005) but not in study 2 (Table 2).

**Table 2:** Changes in cardiometabolic risk measures

	Study 1			Study 2		
	Tirzepatide	Placebo	ETD	Tirzepatide	Placebo	ETD
Change in SBP (mmHg) from baseline at week 48	−9.6 ± 1.1***	−1.7 ± 1.1	−7.9 (−11.0 to −4.9)*** †	−7.6 ± 1.1***	−3.3 ± 1.2**	−4.3 (−7.3 to −1.2)** †
Change in DBP (mmHg) from baseline at week 48	−5.2 ± 0.8***	−2.0 ± 0.8*	−3.2 (−5.4 to −1.0)** †	−3.0 ± 0.7***	−1.8 ± 0.8*	−1.2 (−3.4 to 0.9) †
Percent change in hsCRP from baseline at week 52	−44.2 ± 4.4***	−21.4 ± 6.6**	−28.9 ± 8.2**	−50.7 ± 4.8***	−10.4 ± 9.8	−45.1 ± 8.0***
Percent change in HDL-C from baseline at week 52	10.6 ± 1.5***	3.1 ± 1.5*	7.2 ± 2.1***	15.0 ± 2.0***	4.5 ± 2.0*	10.0 ± 2.8***
Percent change in non-HDL-C from baseline at week 52	−15.0 ± 2.1***	−2.3 ± 2.5	−13.0 ± 3.1***	−15.8 ± 1.6***	−1.8 ± 2.1	−14.3 ± 2.5***
Percent change in triglycerides from baseline at week 52	−32.9 ± 2.6***	−1.0 ± 4.0	−32.2 ± 3.8***	−35.2 ± 2.3***	−5.4 ± 3.8	−31.5 ± 3.7***
Percent change in LDL-C from baseline at week 52	−8.9 ± 2.7**	−4.3 ± 3.0	−4.8 ± 4.2	−10.2 ± 2.0***	0.0 ± 2.5	−10.3 ± 3.0**
Percent change in VLDL-C from baseline at week 52	−32.4 ± 2.5***	0.3 ± 3.9	−32.6 ± 3.7***	−34.9 ± 2.3***	−5.0 ± 3.8	−31.4 ± 3.7***
Percent change in fasting insulin from baseline at week 52	−44.2 ± 3.3***	−4.7 ± 5.9	−41.4 ± 5.0***	−48.5 ± 3.4***	−5.6 ± 6.9	−45.4 ± 5.4***
Percent change in HOMA-IR from baseline at week 52	−51.2 ± 3.2***	−6.2 ± 6.4	−48.0 ± 4.9***	−55.8 ± 3.3***	−2.7 ± 7.9	−54.5 ± 5.0***

The data are an estimate ± standard error.

†The data are the least-squares mean change difference (95% CI).

Mixed model repeated measures were used to analyze continuous variables collected at baseline and more than 1 postbaseline visit. Significance tests were based on least-squares means and type III tests. All hypotheses were tested at a two-sided alpha level of 0.05 for statistical significance, and no adjustments were made for multiplicity.

*P < 0.05, **P < 0.01, ***P < 0.001, representing significant change from baseline within treatment arms or significant ETD between tirzepatide and placebo.

The ETD between tirzepatide and placebo for percent change in hsCRP from baseline at week 52 was significant both in study 1 (−28.9% ± 8.2%; *P* = 0.003) and in study 2 (−45.1% ± 8.0%; *P* <0.001) (Table 2).

The ETD between tirzepatide and placebo for percent change in HDL-C from baseline at week 52 was significant both in study 1 (7.2% ± 2.1%; *P* < 0.001) and in study 2 (10.0% ± 2.8%; *P* < 0.001). The ETD between tirzepatide and placebo for percent change in non-HDL-C from baseline at week 52 was significant both in study 1 (−13.0% ± 3.1%; *P* < 0.001) and in study 2 (−14.3% ± 2.5%; *P* < 0.001). The ETD between tirzepatide and placebo for percent change in triglycerides from baseline at week 52 was significant both in study 1 (−32.2% ± 3.8%; *P* < 0.001) and in study 2 (−31.5% ± 3.7%; *P* < 0.001) (Table 2).

The ETD between tirzepatide and placebo for percent change in LDL-C from baseline at week 52 was not significant in study 1 but was significant in study 2 (−10.3% ± 3.0%; *P* = 0.001). The ETD between tirzepatide and placebo for percent change in VLDL-C from baseline at week 52 was significant both in study 1 (−32.6% ± 3.7%; *P* < 0.001) and in study 2 (−31.4% ± 3.7%; *P* < 0.001) (Table 2).

The ETD between tirzepatide and placebo for percent change in fasting insulin from baseline at week 52 was significant both in study 1 (−41.4% ± 5.0%; *P* < 0.001) and in study 2 (−45.4% ± 5.4%; *P* < 0.001). The ETD between tirzepatide and placebo for percent change in HOMA-IR from baseline at week 52 was significant both in study 1 (−48.0% ± 4.9%; *P* < 0.001) and in study 2 (−54.5% ± 5.0%; *P* < 0.001) (Table 2).

### Mediation analysis

Mediation analysis was performed to estimate the proportion of tirzepatide treatment-associated changes in cardiometabolic risk measures due to either an effect of weight reduction or an effect of changes in sleep-disordered breathing measured by AHI and SASHB.

**Fig. 2:**
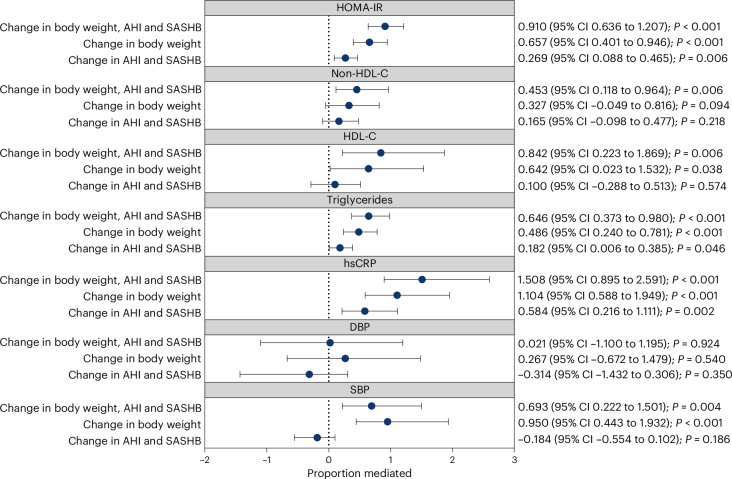
Proportion of tirzepatide effect on cardiometabolic risk measures mediated by body weight, AHI and SASHB. The data are presented as model-based estimate for the mean (95% CI). Statistical analyses were performed using the bootstrap method. All hypothesis testing was conducted using a two-sided framework, and the results are summarized with 95% CIs, which were derived from the corresponding 2.5th and 97.5th percentiles of the bootstrapped estimates (HOMA-IR *n* = 336; non-HDL-C *n* = 341; HDL-C *n* = 342; triglycerides *n* = 341; hsCRP *n* = 342; DBP *n* = 364; SBP *n* = 364). *P < 0.05, **P < 0.01, ***P < 0.001 representing significant proportion mediated.

In a pooled study 1 and study 2 population, the tirzepatide effect on SBP was significantly mediated by combined effect of changes in body weight, AHI and SASHB, and by change in body weight alone. However, the proportion mediated by changes in OSA metrics, AHI and SASHB alone, was not statistically significant (Fig. 2).

The proportions of tirzepatide-associated change in DBP mediated by combined effect of changes in body weight, AHI and SASHB or effects of any of the mediators alone were not statistically significant (Fig. 2).

The tirzepatide-associated change in hsCRP was significantly mediated by combined effect of changes in body weight, AHI and SASHB, by change in body weight alone and by changes in OSA metrics AHI and SASHB (Fig. 2).

The tirzepatide-associated change in triglycerides was significantly mediated by combined effect of changes in body weight, AHI and SASHB, by change in body weight alone and by changes in OSA metrics AHI and SASHB (Fig. 2).

The tirzepatide-associated change in HDL-C was significantly mediated by combined effect of changes in body weight, AHI and SASHB and by change in body weight alone. However, the proportion mediated by change in OSA metrics AHI and SASHB was not statistically significant (Fig. 2).

The tirzepatide-associated change in non-HDL-C was significantly mediated by combined effect of changes in body weight, AHI and SASHB. However, the proportions mediated by change in body weight alone, or mediated by change in OSA metrics, AHI and SASHB, were not statistically significant (Fig. 2).

The tirzepatide-associated change in HOMA-IR was significantly mediated by combined effect of changes in body weight, AHI and SASHB, by change in body weight alone and by change in OSA metrics AHI and SASHB (Fig. 2).

## Discussion

In this study, we observe marked improvements in cardiometabolic risk measures with tirzepatide therapy compared with placebo. These included previously reported improvements in SBP, DBP and hsCRP^13^, as well as improvements in the lipid profile, fasting insulin and HOMA-IR. The improvements in cardiometabolic risk measures were both statistically significant and clinically meaningful and, therefore, tirzepatide treatment may lead to improvement in overall cardiometabolic risk in patients with OSA and obesity. The mediation analysis determined that changes in AHI and SASHB significantly mediated reductions in hsCRP, HOMA-IR and triglycerides, but notably, there was no positive mediation of AHI and SASHB observed on SBP and DBP.

The optimal treatment for patients with OSA and obesity has been unclear. Chirinos et al. reported a comparison of the impact of PAP therapy versus lifestyle weight-loss intervention versus the combination of both treatment approaches^7^. The results, in aggregate, support that treating both OSA as well as obesity may be the optimal treatment, with the awareness and acknowledgment that weight reduction in and of itself mitigates OSA. Of note, randomized trials so far have not demonstrated significant effect of PAP therapy on major cardiovascular outcomes^6,15–19^. The reasons for these findings are unclear, but several potential explanations have been proposed. First, adherence to PAP therapy can be quite variable with major improvements expected only in those with excellent adherence to PAP therapy. Indeed, a recent meta-analysis reported improved cardiometabolic outcomes in patients with OSA who had optimal PAP adherence^20^. Second, the stratification of patients with OSA for participation in randomized controlled trials may be needed to account for cardiometabolic risk. Cluster analyses have suggested that only a subset of patients with OSA may be at major cardiometabolic risk, and thus, randomized controlled trials may need to identify high risk patients a priori rather than the typical one-size-fits-all approach^21,22^. Ongoing efforts using endotyping techniques may help to identify patients most likely to benefit from various interventions in the design of future randomized trials^23^. Third, the lack of observed improvement in outcomes in randomized trials of PAP therapy for OSA in the light of success of bariatric surgery in improving cardiovascular outcomes^24^ may also reflect the failure of some trials to address underlying causes of OSA, such as obesity. In fact, some randomized studies have shown small but significant increases in body weight with PAP therapy, which emphasizes the need to address both OSA and obesity rather than either condition alone^25^. The results from our analyses of SURMOUNT-OSA provide a compelling rationale that improvements in OSA metrics appear to be associated with benefits in addition to those observed by weight reduction. We observed statistically significant independently mediated reductions by OSA metrics in hsCRP, HOMA-IR and triglycerides. In addition, the combination of weight with OSA metrics, but not weight or OSA improvements alone, significantly mediated reductions in non-HDL cholesterol. Of note, the mediation effect of weight on SBP appears to be larger than that for weight and OSA combined, and AHI/SASHB did not show any mediation effect alone. None of our variables were significant mediators of the improvement seen in DBP, suggesting that there may be other unrecognized variables contributing to cardiometabolic benefit with tirzepatide therapy for comorbid obesity and OSA. The implication for clinical practice is that treatment of OSA in patients with OSA and obesity should always include obesity treatment, and medications such as tirzepatide may represent a therapeutic option for such patients. Therapy of OSA in patients with obesity may be initiated by PAP and tirzepatide, with evaluation of PAP therapy continuation being done based on the treatment effect of tirzepatide therapy^26^.

Notably, the finding that the observed improvement in SBP with tirzepatide therapy was significantly mediated only via obesity or the combined effect of body mass index/AHI/SASHB changes but not by independent improvement in OSA was surprising, given the extensive literature on the impact of OSA on SBP. Considerable data from large epidemiological studies and human interventional studies have shown a modest but significant impact of OSA on SBP^27–30^. One potential explanation may be that the point estimate for weight may have less variance than the estimate for AHI, given the night-to-night variability in sleep apnea^31,32^. Indeed, a recent study suggested that multinight recordings of sleep apnea were required to capture a large percentage of the variance in blood pressure^33^. Though study 1 and study 2 were not designed for direct comparison between PAP and non-PAP participants, the results indicate that for participants in study 2, who were on PAP, the improvement in OSA with tirzepatide did not yield the same benefits on both SBP and DBP as for participants who were not on PAP, as PAP may have already improved SBP at baseline. Thus, the findings should not weaken the perception of an important role of OSA treatment in blood pressure control confirmed by previous studies^34,35^.

The mediation analysis compared the effect of tirzepatide on cardiometabolic risk factors through its impact on weight and on OSA severity without reflecting on a possible direct effect of tirzepatide on these factors. The metabolic effect of GLP-1 and GLP-1/GIP receptor agonists appears to integrate their direct effect on pancreatic islet function with long-term weight reduction that increases insulin sensitivity and reduces beta-cell workload in persons with prediabetes and obesity^36^. Animal models have shown that GLP-1 receptor agonists may ameliorate excessive sympathetic activity in the hypertensive-diabetic condition^37^. In addition, GIP receptor agonism, specific for tirzepatide, has been shown to have a direct effect on insulin sensitivity and lipid metabolism^38^. Future research is needed to explore direct effects of tirzepatide on cardiometabolic risk in the realms of OSA as well as mechanisms of its effect on AHI, SASHB and other measures attributed to OSA.

The studies and analysis have a number of strengths. The studies bring results of prespecified endpoints from prospective, controlled, randomized trials. The existing evidence of the relationship between OSA and CVD is largely based on retrospective analyses and, therefore, prospective randomized trial data are necessary to prove some of the concepts created. The mediation analysis of the pooled data from these prospective studies investigates how weight reduction and OSA alleviation improved cardiometabolic risk measures independently, controlling for confounders. This analysis has also provided the size of these effects, and the findings may represent important information for clinical decision making on therapy of patients with OSA and obesity. Despite the strengths of the studies, we acknowledge a number of limitations. First, the studies did not have sufficient duration of follow-up or statistical power to examine the impact of tirzepatide on cardiometabolic outcomes such as myocardial infarction or stroke. Larger studies with longer follow-up may be required to prove that tirzepatide has benefits on these outcomes of interest in patients with OSA and obesity. Second, the SURMOUNT-OSA study excluded people with mild OSA, as well as those with diabetes, and with body mass index <30 kg m^−^^2^. Emerging data suggest that treatment with tirzepatide or GLP-1 receptor agonists results in less weight reduction in patients with type 2 diabetes than in those without diabetes^12,39^. More data are required to determine which patients with OSA are most likely to benefit from tirzepatide.

In SURMOUNT-OSA, tirzepatide mitigated cardiometabolic risk for patients with moderate-to-severe OSA and obesity. Presented results provide support for the concept that treating both OSA and obesity in patients with a combination of both diseases is required to optimize cardiometabolic benefits. Further work is required to determine which patients with OSA benefit the most from different therapeutic approaches.

## Methods

### Study design, procedures and participants

The SURMOUNT-OSA program included two 52-week, randomized, placebo-controlled phase 3 studies. Study 1 included participants who were unwilling or unable to use PAP therapy, and study 2 included participants who had been using PAP therapy for at least 3 months at screening, and who planned to continue PAP for the duration of the trial. Participants were randomly assigned in a 1:1 ratio to receive either tirzepatide at the maximum tolerated dose (10 or 15 mg) or placebo once weekly. The SURMOUNT-OSA program was registered at ClinicalTrials.gov (NCT05412004), and the two studies’ full design, key eligibility criteria, procedures and primary efficacy and safety results have been published previously^13,14^. Participants were screened and enrolled irrespective of their sex. Sex was self-reported by participants.

### Objectives

First, this analysis investigated the association of tirzepatide with changes in cardiometabolic risk markers in participants with moderate-to-severe OSA and obesity. Second, the analysis estimated the relative contribution of weight loss, AHI and SASHB reduction to the improvement in cardiometabolic characteristics of subjects treated with tirzepatide.

Parameters measured included change from baseline in SBP, DBP and levels of hsCRP, HDL-C, non-HDL-C, triglycerides, LDL-C, VLDL-C, fasting insulin and HOMA-IR, in tirzepatide versus placebo.

Mediation analysis was performed to estimate the proportion of tirzepatide treatment-associated changes in cardiometabolic risk measures due to either an effect of weight reduction or an effect of changes in sleep-disordered breathing measured by AHI and SASHB. SASHB is a cumulative measure of oxygen desaturation associated with apneas and hypopneas which, in observational studies, predicted OSA-related cardiovascular mortality and morbidity better than AHI^40,41^.

For each cardiometabolic risk measure, three analyses were performed: estimation of the proportion of efficacy mediated by change from baseline in body weight, change from baseline in AHI and SASHB and, lastly, change from baseline in all three mediators combined.

Improvements in these mediators with tirzepatide treatment compared with placebo from SURMOUNT-OSA studies have been previously published and are as follows:

ETD between tirzepatide and placebo in study 1 was −16.1% (95% CI −18.0 to −14.2) for body weight, −20.0 events per hour (95% CI −25.8 to −14.2) for AHI and −70.1%min h^−1^ (95% CI −90.9 to −49.3) for SASHB^13^. ETD between tirzepatide and placebo in study 2 was −17.3% (95% CI −19.3 to −15.3) for body weight, −23.8 events per hour (95% CI −29.6 to −17.9) for AHI and −61.3%min h^−1^ (95% CI −84.7 to −37.9) for SASHB^13^.

### Statistical analysis

Changes in SBP, DBP and hsCRP from SURMOUNT-OSA have already been reported using treatment-regimen estimand analysis^13^. The analyses reported here were guided by the ‘efficacy’ estimand; the analysis included data collected before permanent discontinuation of study intervention and was conducted using the efficacy analysis set. For efficacy analysis, missing values were imputed using multiple imputation based on the reason of intercurrent events. All results are reported using the efficacy estimand unless otherwise specified.

Unless otherwise noted, all tests of treatment effects were conducted at a two-sided alpha level of 0.05, and the CIs were calculated at 95%. In statistical summaries and analyses, participants were analyzed as randomized.

The mixed model repeated measures analysis, a restricted-maximum-likelihood-based model, was used to analyze continuous longitudinal variables. All the longitudinal observations at each scheduled postbaseline visit were included in the analysis. The model includes the fixed class effects of treatment, strata (pooled country/geographic region, baseline OSA severity and gender), visit and treatment-by-visit interaction, as well as the continuous, fixed covariate of baseline value. The significance tests were based on least-squares means and type III tests. As all of these endpoints were exploratory in nature, the *P* values were not adjusted for multiplicity. Statistical analyses were performed using SAS version 9.3.

Mediation analyses were conducted to decompose the total effect of tirzepatide on cardiometabolic risk markers into direct effect and indirect effect. The direct effect quantified the effect of tirzepatide versus placebo on change in cardiometabolic outcomes, independent of changes in mediators. Conversely, the indirect effect captured the effect of tirzepatide versus placebo on change in cardiometabolic outcomes associated with changes in mediators. Because these comparisons involve non-observable counterfactual scenarios, natural effect models were employed to estimate the direct and indirect effects^42^. The total effect was defined as the sum of these two components. The proportion mediated was calculated as the indirect effect divided by the total effect then multiplying by 100%, and the standard errors were derived using the bootstrap method. The mediation analyses were performed on the pooled study 1 and study 2 populations. The outcome was the change in cardiometabolic risk measure from baseline to the end of the study. Analyses on SBP and DBP were performed on the original scale, and analyses on hsCRP, HDL-C, non-HDL-C, triglycerides and HOMA-IR were performed on the log scale. Baseline covariates included region, sex, baseline cardiometabolic risk measure, AHI, body weight and log-transformed SASHB. We investigated the effects of mediators for three scenarios: first, a single mediator of change from baseline in body weight; second, two independent mediators of change from baseline in AHI and change from baseline in SASHB to represent changes attributable to OSA severity improvement; and lastly, a combination of the effect of changes in body weight, AHI and SASHB as three parallel mediators. The post-treatment confounders were controlled for and included change in AHI and SASHB when the mediator was change from baseline in body weight and change in body weight when the mediator was change from baseline in AHI and SASHB. No post-treatment confounders were controlled for when all of the change from baseline in body weight, AHI and SASHB were mediators. Log transformation was always applied for SASHB. The mediation analyses were performed using the ‘CMAverse’ package version 0.1.0 in R version 4.1.2 (ref. ^42^).

Pearson’s correlation coefficients were also calculated between the cardiometabolic risk measures and the sleep-disordered breathing parameters on the pooled study 1 and study 2 populations. Analyses on SBP and DBP were performed on the original scale, and analyses on hsCRP, HDL-C, non-HDL-C, triglycerides and HOMA-IR were performed on the log scale. These results are presented in Extended Data Table 1.

### Ethics and informed consent

The study was approved by ethics review boards at each site, presented in Supplementary Table 1.

This study was conducted in accordance with consensus ethical principles derived from international guidelines including the Declaration of Helsinki and Council for International Organizations of Medical Sciences International Ethics Guidelines, applicable ICH GCP Guidelines, International Organization for Standardization (ISO) 14155 and applicable laws and regulations.

Participants or their legally authorized representatives signed a statement of informed consent that meets the requirements of 21 Code of Federal Regulations 50, local regulations, ICH guidelines, privacy and data protection requirements, where applicable, and the IRB/IEC or study center.

### Reporting summary

Further information on research design is available in the Nature Portfolio Reporting Summary linked to this Article.

## Online content

Any methods, additional references, Nature Portfolio reporting summaries, source data, extended data, supplementary information, acknowledgements, peer review information; details of author contributions and competing interests; and statements of data and code availability are available at 10.1038/s41591-025-04071-1.

## Supplementary information

Supplementary Information Supplementary Table 1.

Reporting Summary

## Source data

Source Data Fig. 1 Mediation analyses.

## Figures and Tables

**Extended Data Table 1:**
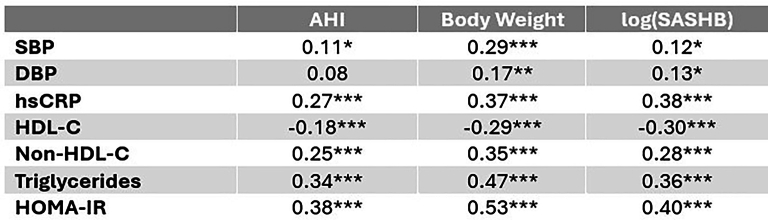
Correlation between change in cardiometabolic parameters and change in body weight, AHI or SASHB

*p-value < 0.05, **p-value < 0.01, ***p-value < 0.001 representing significant Pearson’s correlation coefficient.

## Data Availability

Data from the analyses in this study cannot be shared publicly due to the sponsor’s (Eli Lilly and Company) contractual obligations. Lilly provides access to all individual participant data collected during the trial, after anonymization, with the exception of pharmacokinetic or genetic data. Data are available to request 6 months after the indication studied has been approved in the USA and European Union and after primary publication acceptance, whichever is later. No expiration date of data requests is currently set once data are made available. Access is provided after a proposal has been approved by an independent review committee identified for this purpose and after receipt of a signed data sharing agreement. Data and documents, including the clinical study report, blank or annotated case report forms, will be provided in a secure data sharing environment. For details on submitting a request, see the instructions provided at www.vivli.org. Contact the corresponding author for details on submitting a request. Source data are provided with this paper.

## Code availability

No novel code was used for data analysis in the current study.
