# Supplementary material for: Tirzepatide on obstructive sleep apnea-related cardiometabolic risk: secondary outcomes of the SURMOUNT-OSA randomized trial
Source: Nat Med. 2026 Jan 15;32(2):653–9. doi: 10.1038/s41591-025-04071-1 (PMC12920140; doi:10.1038/s41591-025-04071-1)
Supplement: Supplementary file 1 — Supplementary Table 1. [file 41591_2025_4071_MOESM1_ESM.pdf]

nature medicine

Supplementary information https://doi.org/10.1038/s41591-025-04071-1

Tirzepatide on obstructive sleep apnea-related cardiometabolic risk: secondary outcomes of the SURMOUNT-OSA randomized trial

In the format provided by the authors and unedited

Table of Contents | **Page**

Table S1. Ethics review boards by site country	2

Supplementary Table 1: Ethics review boards by site country

Site Country	Ethics Review Board
Australia	Bellberry Health and Research Ethics
	Adelaide Institute for Sleep Health
	Woolcock Institute of Medical Research (Internal Governance)
Brazil	Hospital de Clinicas de Porto Alegre
	Investiga
	Comissao de Etica para Analise Projetos de Pesquisa-CAPPesq
	ISBEM – Instituto de Saúde e Bem-Estar da Mulher
	Fundação Faculdade de Medicina do ABC
	Instituto de Pesquisa clinica de Campinas
China	Clinical Trial Ethics Committee of West China Hospital, Sichuan University
	Drug Clinical Ethics Committee of Tianjin Medical University General Hospital
	The EC of Zhongshan Hospital affiliated to Fudan University
	The First Hospital of Jilin University
	Beijing Hospital
	Wuxi People’s Hospital
	The Second Affiliated Hospital of Nanjing Medical University
Czech Republic	Etick Komise IKEM a Thomayerovy Nemocnice
Germany	Ethikkommissionen bei der Ärztekammer Schleswig-Holstein
	Landesamt für Gesundheit und Soziales (LAGeSO)
	Ethikkommission der Ärztekammer Hamburg
	Ethikkommission bei der Schleswig-Holstein
	Ethikkommission Ärztekammer Nordrhein
	Ethikkommission bei der Ärztekammer Niedersachsen
	Ethikkommission der Ärztekammer Westfalen-Lippe und der Medizinischen
Japan	AMC Nishiumeda Clinic
	Sakai City Medical Center
	Review Board of Human Rights and Ethics for Clinical Studies
	Kojunkai Daido Clinic
	Tokyo-Eki Center-building Clinic Institutional Review Board
	Hayashi Diabetes Internal Medicine Clinic
	Osaka Kaisei Hospital
Mexico	Unidad de Investigacion en Salud de Chihuahua
	RM Pharma Specialists
	Investigación Biomédica para el Desarrollo de Fármacos
	Hospital Hispano
	Investigación Biomédica para el Desarrollo de Fármacos S.A. de C.V.
	Centro Especializado en Diabetes Obesidad Y Prevencion de Enfermedades Cardiovasculares S.C.
	Comite RM PHARMA SPECIALISTS S.A. DE C.V.
Taiwan	Institutional Review Board, National Cheng Kung University Hospital
	Institutional Review Board, China Medical University Hospital
United States	Advarra Inc.
