# Supplementary material for: Tirzepatide on obstructive sleep apnea-related cardiometabolic risk: secondary outcomes of the SURMOUNT-OSA randomized trial
Source: Nat Med. 2026 Jan 15;32(2):653–9. doi: 10.1038/s41591-025-04071-1 (PMC12920140; doi:10.1038/s41591-025-04071-1)
Supplement: Supplementary file 2 — Reporting Summary [file 41591_2025_4071_MOESM2_ESM.pdf]

nature portfolio

Corresponding author(s): Josef Bednarik

Last updated by author(s): Sep 26, 2025

Reporting Summary

Nature Portfolio wishes to improve the reproducibility of the work that we publish. This form provides structure for consistency and transparency in reporting. For further information on Nature Portfolio policies, see our Editorial Policies and the Editorial Policy Checklist.

Statistics

For all statistical analyses, confirm that the following items are present in the figure legend, table legend, main text, or Methods section.

n/a	Confirmed	
☐	☒	The exact sample size (*n*) for each experimental group/condition, given as a discrete number and unit of measurement
☒	☐	A statement on whether measurements were taken from distinct samples or whether the same sample was measured repeatedly
☐	☒	The statistical test(s) used AND whether they are one- or two-sided<br>*Only common tests should be described solely by name; describe more complex techniques in the Methods section.*
☐	☒	A description of all covariates tested
☐	☒	A description of any assumptions or corrections, such as tests of normality and adjustment for multiple comparisons
☐	☒	A full description of the statistical parameters including central tendency (e.g. means) or other basic estimates (e.g. regression coefficient) AND variation (e.g. standard deviation) or associated estimates of uncertainty (e.g. confidence intervals)
☐	☒	For null hypothesis testing, the test statistic (e.g. *F*, *t*, *r*) with confidence intervals, effect sizes, degrees of freedom and *P* value noted<br>*Give P values as exact values whenever suitable.*
☒	☐	For Bayesian analysis, information on the choice of priors and Markov chain Monte Carlo settings
☒	☐	For hierarchical and complex designs, identification of the appropriate level for tests and full reporting of outcomes
☐	☒	Estimates of effect sizes (e.g. Cohen's *d*, Pearson's *r*), indicating how they were calculated

Our web collection on statistics for biologists contains articles on many of the points above.

Software and code

Policy information about availability of computer code

Data collection	No novel code was used for data collection
Data analysis	No novel code was used for data analysis in the current study. Statistical analyses were performed using SAS (version 9.3). The mediation analyses were performed using the 'CMAverse' package Version 0.1.0 in R Version 4.1.2.

For manuscripts utilizing custom algorithms or software that are central to the research but not yet described in published literature, software must be made available to editors and reviewers. We strongly encourage code deposition in a community repository (e.g. GitHub). See the Nature Portfolio guidelines for submitting code & software for further information.

Data

Policy information about availability of data

All manuscripts must include a data availability statement. This statement should provide the following information, where applicable:

- Accession codes, unique identifiers, or web links for publicly available datasets
- A description of any restrictions on data availability
- For clinical datasets or third party data, please ensure that the statement adheres to our policy

Data from the analyses in this study cannot be shared publicly due to the sponsor's (Eli Lilly and Company) contractual obligations. Lilly provides access to all individual participant data collected during the trial, after anonymization, with the exception of pharmacokinetic or genetic data. Data are available to request 6 months after the indication studied has been approved in the US and EU and after primary publication acceptance, whichever is later. No expiration date of data

requests is currently set once data are made available. Access is provided after a proposal has been approved by an independent review committee identified for this purpose and after receipt of a signed data sharing agreement. Data and documents, including the study protocol, statistical analysis plan, clinical study report, blank or annotated case report forms, will be provided in a secure data sharing environment. For details on submitting a request, see the instructions provided at www.vivli.org. Contact the corresponding author for details on submitting a request. Source data are provided with this paper.

Research involving human participants, their data, or biological material

Policy information about studies with human participants or human data. See also policy information about sex, gender (identity/presentation), and sexual orientation and race, ethnicity and racism.

Reporting on sex and gender	Participants were screened and enrolled irrespective of their sex. Sex was self reported by participants. Data are provided in Table 1.
Reporting on race, ethnicity, or other socially relevant groupings	Participants were screened and enrolled irrespective of their race and ethnicity. Race and ethnicity were self-reported by participants.
Population characteristics	Participant characteristics are provided in Table 1 and include age, gender, BMI, AHI, OSA severity, SASHB, HbA1c, hypertension, prediabetes.
Recruitment	The participants were enrolled using the predefined inclusion/exclusion criteria, which have been previously published.
Ethics oversight	The study was conducted in accordance with international guidelines including the Declaration of Helsinki and Council for International Organizations of Medical Sciences International Ethical Guidelines. All participants provided written informed consent. The names of the boards/committees that approved the protocol are as follows: Bellberry Health and Research Ethics, Adelaide Institute for Sleep Health, Woolcock Institute of Medical Research (Internal Governance), Hospital de Clinicas de Porto Alegre, Investiga, Comissao de Etica para Analise Projetos de Pesquisa-CAPPesq, ISBEM – Instituto de Saúde e Bem-Estar da Mulher, Fundação Faculdade de Medicina do ABC, Instituto de Pesquisa clinica de Campinas, Clinical Trial Ethics Committee of West China Hospital, Sichuan University, Drug Clinical Ethics Committee of Tianjin Medical University General Hospital, The EC of Zhongshan Hospital affiliated to Fudan University, The First Hospital of Jilin University, Beijing Hospital, Wuxi People's Hospital, The Second Affiliated Hospital of Nanjing Medical University, Etick Komise IKEM a Thomayerovy Nemocnice, Ethikkommissionen bei der Ärztekammer Schleswig-Holstein, Landesamt für Gesundheit und Soziales (LAGeSO), Ethikkommission der Ärztekammer Hamburg, Ethikkommission bei der Schleswig-Holstein, Ethikkommission Ärztekammer Nordrhein, Ethikkommission bei der Ärztekammer Niedersachsen, Ethikkommission der Ärztekammer Westfalen-Lippe und der Medizinischen, AMC Nishiumeda Clinic, Sakai City Medical Center, Review Board of Human Rights and Ethics for Clinical Studies, Kojunkai Daido Clinic, Tokyo-Eki Center-building Clinic Institutional Review Board, Hayashi Diabetes Internal Medicine Clinic, Osaka Kaisei Hospital, Unidad de Investigacion en Salud de Chihuahua, RM Pharma Specialists, Investigación Biomédica para el Desarrollo de Fármacos, Hospital Hispano, Investigación Biomédica para el Desarrollo de Fármacos S.A. de C.V., Centro Especializado en Diabetes Obesidad Y Prevencion de Enfermedades Cardiovasculares S.C., Comite RM PHARMA SPECIALISTS S.A. DE C.V., Institutional Review Board, National Cheng Kung University Hospital, Institutional Review Board, China Medical University Hospital, Advarra Inc.

Note that full information on the approval of the study protocol must also be provided in the manuscript.

Field-specific reporting

Please select the one below that is the best fit for your research. If you are not sure, read the appropriate sections before making your selection.

☒ Life sciences ☐ Behavioural & social sciences ☐ Ecological, evolutionary & environmental sciences

For a reference copy of the document with all sections, see nature.com/documents/nr-reporting-summary-flat.pdf

Life sciences study design

All studies must disclose on these points even when the disclosure is negative.

Sample size	Approximately 206 participants per ISA were planned to be randomized to either tirzepatide or placebo.
Data exclusions	The statistical analysis plan defined population as adult participants with obesity and OSA who received at least one dose of study treatment.
Replication	As this was a clinical study, measurements were not replicated for each patient. Sufficient number of participants were included to obtain appropriate population means.
Randomization	Patients were randomized 1:1 to receive either tirzepatide or placebo.
Blinding	This is a double-blind study in which participants and study personnel, are blinded to study intervention.

Reporting for specific materials, systems and methods

We require information from authors about some types of materials, experimental systems and methods used in many studies. Here, indicate whether each material, system or method listed is relevant to your study. If you are not sure if a list item applies to your research, read the appropriate section before selecting a response.

Materials & experimental systems

n/a	Involved in the study
☒	☐ Antibodies
☒	☐ Eukaryotic cell lines
☒	☐ Palaeontology and archaeology
☒	☐ Animals and other organisms
☐	☒ Clinical data
☒	☐ Dual use research of concern
☒	☐ Plants

Methods

n/a	Involved in the study
☒	☐ ChIP-seq
☒	☐ Flow cytometry
☒	☐ MRI-based neuroimaging

Clinical data

Policy information about clinical studies

All manuscripts should comply with the ICMJE guidelines for publication of clinical research and a completed CONSORT checklist must be included with all submissions.

Clinical trial registration	This trial is registered with ClinicalTrials.gov, NCT05412004
Study protocol	Previously published (DOI:10.1056/NEJMoa2404881) and included with submission.
Data collection	Data was collected at 58 locations in the United States, Australia, Brazil, China, Czechia, Germany, Japan, Mexico, Puerto Rico, and Taiwan. The full list of each individual site can be found at ClinicalTrials.gov, NCT05412004. The study started on 21/06/2022 and was completed on 29/03/2024.
Outcomes	We include the previously reported prespecified secondary outcomes including changes in inflammatory status (measured via hsCRP), and blood pressure (systolic and diastolic), measured up to week 52. Additional, prespecified secondary outcomes were cardiometabolic risk measures including HDL-C, non-HDL-C, and fasting insulin measured up to week 52. Post-hoc analyses were change in HOMA-IR measured up to week 52, and proportion of observed changes in cardiometabolic risk measures attributable to reductions in body weight, AHI, and SASHB (assessed by mediation analysis and described fully in the Methods section).

Plants

Seed stocks	*Report on the source of all seed stocks or other plant material used. If applicable, state the seed stock centre and catalogue number. If plant specimens were collected from the field, describe the collection location, date and sampling procedures.*
Novel plant genotypes	*Describe the methods by which all novel plant genotypes were produced. This includes those generated by transgenic approaches, gene editing, chemical/radiation-based mutagenesis and hybridization. For transgenic lines, describe the transformation method, the number of independent lines analyzed and the generation upon which experiments were performed. For gene-edited lines, describe the editor used, the endogenous sequence targeted for editing, the targeting guide RNA sequence (if applicable) and how the editor was applied.*
Authentication	*Describe any authentication procedures for each seed stock used or novel genotype generated. Describe any experiments used to assess the effect of a mutation and, where applicable, how potential secondary effects (e.g. second site T-DNA insertions, mosiacism, off-target gene editing) were examined.*
